# Hotel Dieu

**Published:** 1830-10

**Authors:** 


### HOTEL DIEU.

### *Foreign Body in the Trachea; Tracheotomy; with Practical Observations on the Operation.* By M. Dupuytren.†

A child, eight years of age, having been sent on an errand by her mother, was tempted by the sight of a kidney bean, which was lying in a grocer's shop. She snatched it up, and put it quickly in her mouth to conceal it. She did this precipitately, and at the moment of inspiring deeply, so that the bean entered the larynx and passed into the trachea, where it remained. Violent cough and paroxysms of suffocation immediately took place. The

† Journal Hebdomadaire, &c. No. 80.

accident happened on the 13th February, 1830. Several physicians were called in, and an emetic was given, which produced severe vomiting, but did not cause the expulsion of the foreign body. During the remainder of the day, the little patient was sometimes free from suffering, at others threatened with suffocation.

12th. In the evening she was admitted into the Hotel Dieu, under the care of M. Dupuytren. She passed a very restless night. The attacks of suffocation were very violent, and were frequently followed by prolonged insensibility, which placed her in great danger.

In the morning she was much troubled with violent cough. Upon applying the ear to the neck opposite the trachea, a peculiar rustling noise was heard, and the sound of the foreign body in the canal was perceived. The indication was evident. It was necessary to remove the foreign body by making an opening into the aerial passage, and M. Dupuytren determined upon the operation of tracheotomy, which was performed on the 13th, in the following manner. A few minutes before the operation, attempts were again made to hear the shock of the foreign body against the sides of the canal, but it was not now distinct. We shall afterwards refer to this important sign, and also to the cause of its absence at the moment of the operation.

The child was placed upon a bed, and held by assistants, the head being thrown back. An incision, about one inch and a quarter long, was made upon the median line, and a little above the superior edge of the sternum. The skin alone was first divided, and then, very cautiously, the subcutaneous cellular tissue. The muscles were moved on each side, and the trachea was exposed without a single blood-vessel being divided or tied. M. Dupuytren, then, by means of a straight-pointed bistoury, directed upon the nail of the left index finger, divided, in a vertical direction, several of the cartilaginous rings of the trachea and the fibrous membrane which unites them. At first no air escaped, and the voice of the patient remained entirely unaltered, much to the surprise of the surgeon and his assistants. But the incision being enlarged a little upwards and downwards, and the edges of the wound held apart by forceps, air immediately escaped. The child's head was now slightly bent forwards, and air passed out more freely: her voice became more powerful, and, after some violent efforts at expiration, the bean was expelled, covered with bloody mucus, and fell upon the patient's chest. The respiration was now natural; the attacks of suffocation and cough ceased, and the child in fact appeared to be perfectly free from complaint. The edges of the wound were carefully cleansed from the frothy blood which covered them, and were not brought together. A compress of lint, covered with a simple dressing, and with a hole in the centre, was placed upon the fore part of the neck. No lint was laid over it, but merely a fine compress, which was retained by a very loose bandage. The bean was about five lines in length, and three in breadth: it was a little swollen. In the evening the patient was very feverish. V.S. She passed a comfortable night.

14th. Air passed freely through the wound, and respiration appeared to be carried on almost entirely through the artificial opening. From the mucous secretion which obstructed the breathing, it was rather noisy. Pain in the larynx; occasional dyspnœa. Fifteen leeches were applied to the sides of the neck; mild drinks ordered. The leeches drew blood freely, and the patient was much and permanently relieved: but little cough; slept tolerably well; fever abated.

15th. Breathes easily: a slight hissing noise is heard from the wound; skin of a natural heat.

16th and 17th. In a very favorable state: sleep calm and uninterrupted; speech distinct and free. A small quantity of air still passes through the wound, which may be accounted for in a great measure from the swollen state of its edges.

20th. Edges of the wound brought together, as there is no longer any danger of emphysema. A mucous tissue now covers the lips of the wound, and renders the cellular tissue impermeable.

No other symptom occurred of any consequence. In a few days the child was taken home, the wound being reduced to about two thirds its original size. In a month after the operation, she was brought to the hospital: at this period there was still a very small external opening, through which a little air escaped. The wound will doubtless be entirely closed in a few days, and the patient be perfectly cured.

*Observations.* Several interesting remarks were made upon this case by M. Dupuytren.

1st. In relation to the position in which the patient should be placed during the operation, for the purpose of dividing the external soft parts and the trachea; also in regard to the position in which the patient should remain after the trachea has been opened, in order to favor the exit of the foreign body lodged in it. For the division of the soft parts and the trachea, the head should be thrown backwards: but this position is unfavorable for the exit of air from the trachea when an opening is made into it, and still more so for the expulsion of a foreign body. It was found in the above case that, when the incision was made into the trachea, no air passed out, and the foreign body was not expelled. This was probably owing in a great measure to the small extent of the wound, but the backward position of the head had certainly some share in preventing the immediate success of the operation, as the edges of the wound were thus kept upon the stretch, and prevented from separating. On the contrary, when the head is inclined forwards, the edges of the wound separate more easily, and therefore permit the exit of air or a foreign body. In this case the bean was spontaneously expelled when the head was placed in this position. From the circumstances which here happened, the practical precept therefore arises that the head should be thrown backwards during the operation of tracheotomy; but, to facilitate the free issue of air and the spontaneous expulsion of the foreign body, it is necessary to bend the head forwards, in order to produce a separation of the edges of the wound.

Another difficulty which may be met with in the course of the operation, requires attention. There exists, between the sterno-thyroideus and the sterno-hyoideus muscles and the anterior part of the trachea, a space filled with loose cellular tissue, which forms a kind of cavity. When the instruments arrive at this part, if they do not enter the trachea by the opening which has been made in it, they may pass into this cellular cavity, and lead to mistakes. A little care, however, will prevent this accident. We must again refer to one of the characteristic signs which denotes the presence of a foreign body in the trachea: it is that of the sensation of the shock of the body against the parietes of the canal. This sensation may be detected both by the hand and ear.

We are not aware that this very important symptom has been mentioned by any surgeon before M. Dupuytren. It is not so distinct in every case, and may be less perceptible at different periods after the introduction of the foreign body. The foreign body may adhere to the surrounding parts, and then, not being displaced by the air, it does not strike against the parietes of the canal; or, from being enveloped by an abundant and thick mucous secretion, the shock it produces will be much slighter. In the above case the sound was distinct when the patient entered the hospital, but much less so at the time of the operation.

The manner in which the wound was dressed also deserves attention. Its edges were not immediately brought together, that the patient might not be exposed to emphysema. During the first days the cellular tissue would have been permeable, and in all probability would easily have given passage to air; but after this period, when it was hardened and rendered compact by inflammation, this was no longer possible, and the wound was closed without danger. This precaution ought always to be adopted. It is to be observed also that no lint was placed upon the linen with the aperture in it, which was applied to the wound. When lint has been applied, it has sometimes entered the trachea, and produced very serious mischief.

---

### *Foreign Body extracted from the upper Part of the Larynx.*

A WOMAN, about fifty years of age, presented herself at the Hotel Dieu, March 20th, 1830, to obtain relief from the effects of an accident which had happened to her eight or ten days before. While eating fish she swallowed a bone, and immediately afterwards she felt pain in the throat at the top of the larynx. This pain continued, and was troublesome both in the act of swallowing and breathing. At first the inconvenience was but trifling, but the pain gradually became more acute. The patient's mouth was opened as widely as possible, and M. Dupuytren carried his finger to the base of the tongue, as far as the epiglottis, the circumference of which he examined. He felt a projection about the size of a large pin's head: the remainder of the foreign body was fixed under the epiglottis. From the account given by the patient, he presumed it to be a fish bone, but he could not himself determine its nature. M. D., having the index finger of one hand placed upon the projecting body, introduced a pair of forceps along his finger, and was enabled to grasp it, and, by a slight extractile force, he removed a fish bone about one inch and a quarter long. The patient suffered very little pain from the extraction. No hemorrhage followed; and as she did not make her appearance again, she probably remained free from any subsequent inconvenience.

It is remarkable that in this case the foreign body was fixed in the parietes of the larynx, and across its upper extremity. It is also curious that the irritation, which is generally so violent when foreign bodies are lodged in the air-passages, was so very slight in this case, that the patient submitted to it for several days without much inconvenience. It is, in fact, an additional example of the great difference in the sensibility of the same organs in different persons. A foreign body thus situated would generally produce very formidable symptoms.*

